# Sweet Relief? Short-Term Post-Traumatic High-Sucrose Intake Attenuates Acute but Not Long-Term Fear Responses in Mice

**DOI:** 10.3390/biomedicines13092233

**Published:** 2025-09-11

**Authors:** Prabhat Kumar, Pedro Correia, Imola Plangár, Dóra Zelena

**Affiliations:** 1 Institute of Physiology, Medical School, Centre for Neuroscience, Szentágothai Research Centre, University of Pécs, H7624 Pécs, Hungary; prabhatmbcs@gmail.com (P.K.);; 2 János Szentágothai School of Neurosciences, Semmelweis University, H1085 Budapest, Hungary

**Keywords:** post-traumatic stress disorder, acute stress disorder, metabolic disorder, sucrose, fear memory, freezing

## Abstract

People often turn to sweet foods for comfort during times of stress, as energy imbalance is implicated in several neuropsychiatric disorders including post-traumatic stress disorder (PTSD). Although acute sucrose consumption may improve cognitive capabilities, its long-term effectiveness has been debated. **Objectives**: In a widely used mouse model, we examined the effect of sucrose drinking on conditioned fear-induced freezing (as a model of PTSD), with emphasis on the concentrations and timing of the intervention as well as sex differences. We aimed to develop a low-cost, widely accessible therapeutic option. **Methods**: A short electric foot shock was used for trauma, and freezing was detected 24 h (mimicking acute stress disorder, ASD) or 14 days (PTSD-like symptoms) later in the trauma context and with trauma cues. **Results**: First, we confirmed that our trauma increased freezing, independent of previous habituation to sucrose drinking. Next, we confirmed that 16% and 32%, but not 2% sucrose drinking for 24 h (but not 3 h) immediately after trauma, diminished freezing behavior the next day. However, the same intervention did not influence behavior 14 days later. Moreover, we could not find any curative effect of 24 h of 16% sucrose consumption before testing remote fear memory 14 days after trauma. **Conclusions**: Consuming a high-calorie solution immediately following trauma for 24 h may influence ASD but does not necessarily alter the development of PTSD symptoms. Here, we offer a new perspective on energy regulation in neuropsychiatric disorders.

## 1. Introduction

Throughout history and across cultures, people have turned to sweet foods for comfort during stressful times. Sugar-rich foods have long been associated with psychological relief by offering a piece of candy to calm a distressed child to indulge in chocolate after a difficult day. This notion is so deeply ingrained in popular culture that even in Harry Potter, Professor Lupin famously gives Harry chocolate after a Dementor attack, highlighting the almost universal belief in the comforting properties of sweets [1]. However, the belief that sugar intake may offer acute benefits in individuals experiencing post-traumatic stress disorder (PTSD) warrants further investigation into its therapeutic potential.

PTSD can potentially affect individuals worldwide, as people from any country or cultural background may be at risk of developing this disorder. Persistent traumatic memories, frequently accompanied by extreme fear and anxiety, are one of the defining symptoms of PTSD [2]. Thus, effective therapies for PTSD must concentrate on influencing the generation and consolidation of fear memories [3]. Two-thirds of people in Europe have experienced at least one traumatic event during their lifetime, and 1–3% of persons in Europe or roughly 7.7 million people have developed PTSD and are currently experiencing symptoms [4]. With variances in trauma exposure, such as war and battle events or natural disasters, cultural influences, and healthcare support, the prevalence of PTSD varies greatly between European nations [5]. Nevertheless, the COVID-19 pandemic increased the risk of PTSD across Europe; approximately 30% of the population had symptoms consistent with PTSD [6,7,8]. This was an unprecedented challenge for mental health services [9]. Thus, it is reasonable to look for a simple and inexpensive solution, especially for prevention.

Novel PTSD treatment strategies may be developed based on experimental fear memory studies [10,11]; the conditioned fear test (CFT) is one of the best-known and widely accepted animal models of this disorder [12,13,14]. The brain is one of the most energy-demanding organs, as approximately 20% of the calories consumed are devoted to it, with the lion’s share provided in the form of glucose [15]. Acute mental stress increases brain energy needs [16]. In contrast, brain energy metabolism actively regulates synaptic transmission and activity [17]. Chronic stress may jeopardize homeostatic energy balance [15]. Therefore, stress-related psychiatric conditions, including PTSD, are considered metabolic disorders [18]. Investigating the relationship between carbohydrate intake and the mitigation of trauma-related behaviors, particularly freezing behavior, in a mouse model of PTSD may illuminate potential diet-based interventions that could alleviate symptoms [19]. This understanding may reveal a practical, accessible, and non-pharmaceutical treatment that promotes positive mental health outcomes following trauma exposure [20].

On the other hand, in recent decades, sugar consumption has increased dramatically, leading to an increased prevalence of obesity and diabetes. Commonly referred to as table sugar, sucrose is a disaccharide composed of glucose and fructose monosaccharides and is used as a sweetener in many different foods and beverages [21]. It has a definite positive acute cognitive impact owing to its ability to alter neurotransmitter systems, including those involved in the development of fear memories [22,23,24,25]. An American group found that 40% glucose, but not fructose, drinking immediately after trauma was helpful in preventing the development of PTSD-like symptoms in rats [20,26]. They even confirmed blood glucose and liver glycogen elevation and lower corticosterone stress hormone elevation after glucose consumption [20]. However, they used a tail shock–restraint combination and tested the animals 24 h after trauma in an active avoidance paradigm. Both the timing (24 h is more an acute stress disorder (ASD) than PTSD) and the test (active avoidance is a learning parameter) were questionable for modelling PTSD. Moreover, an Argentine group reported that 10% sucrose drinking in juvenile, but not adult rats, impaired fear memory extinction [27] and recognition memory [28] in adulthood. Thus, it has a negative, rather than a positive, long-term effect. Additionally, a meta-analysis of 1259 participants found no positive effect of acute carbohydrate consumption on any aspect of mood at any time-point studied [29]. However, relatively short periods (immediate (0–30 min), short-term (31–60 min), and long-term (61+ min) effects) without stress were examined.

Here, we aimed to clarify whether sucrose consumption might effectively prevent or treat PTSD-like symptoms. Most previous studies have been conducted in rats. However, to reveal the mechanism, transgenic mice would be useful (e.g., a mouse strain containing Cre enzyme in their glucose transporter 2-positive cells, marking the brain glucose sensors [30,31]). Therefore, in addition to generalizations, it is very important to study the effects of sugar consumption in mouse models. Since the transgenic strains were created mostly based on a C57BL/6 background, this strain was the subject of our study. In the experiments leading to the formulation of the comfort food theory (i.e., sucrose can diminish our stress axis during chronic stress, similar to corticosterone) [32], 30% sucrose was used [33,34], with a similar high calorie content as in previous PTSD-related studies (40%) [20,26]. In contrast, anhedonic animals avoid drinking 1% sucrose [35]; however, after chronic social isolation, they might even prefer 32% sucrose compared to group-housed rats [36]. Moreover, there seems to be a peak at 16% sucrose preference in C57BL/6 mice [37]. Therefore, we provided 2–16–32% sucrose immediately after trauma (prevention) or (in some selected cases) before testing 2 weeks later (treatment). We focused our investigation on the freezing response, as it is a central and widely acknowledged outcome of CFTs. This response serves as an indicator of defensive behaviors that are notably modified in animal models of PTSD [13,14,38]. Although PTSD is more prevalent in women [39], most studies were conducted on male subjects. Thus, we also addressed sex differences.

## 2. Materials and Methods

### 2.1. Animals

Adult C57Bl/6 mice of both sexes from our local colony (Pécs University, Pécs, Hungary; original breeding pairs were purchased from Charles River Laboratory, Budapest, Hungary) were used in a balanced design. The mice (242) were separated 2–3 days before the experiments to follow individual fluid consumption. This separation also helped minimize potential confounders, such as the different number of animals in one cage. Additionally, all animals were on the same shelf level. The sample size was based on our previous study using fear conditioning (FC) [40]. The animals were kept in a standard environment (21 ± 1 °C, 12 h light/dark cycle with lights on at 9 p.m.) and had access to food (standard laboratory chow; Charles River, Hungary) and water ad libitum. Behavioral examinations were conducted at the beginning of the dark, active phase under red light, as rodents are nocturnal animals. The animals were randomly assigned to different groups to ensure that treatments were equally distributed between sexes, and animals of approximately the same weight were assigned to different groups. Therefore, automated software was not used for randomization.

### 2.2. Experiment Design

**Figure 1:**
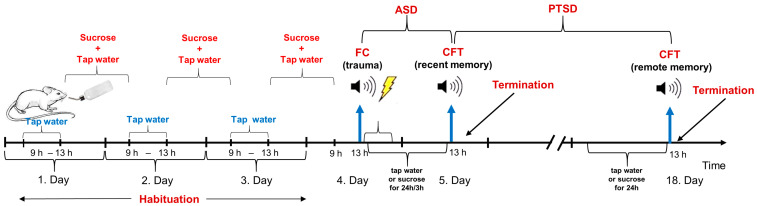
Schematic representation of experimental design. Abbreviations: ASD: acute stress disorder; CFT: conditioned fear test; FC: fear conditioning; PTSD: post-traumatic stress disorder.

Based on previous studies, we used 2%, 16%, and 32% sucrose solutions (20, 160, or 320 g sugar in 1000 mL tap water at room temperature). Although a high sucrose concentration can provide energy, the high osmolality of the solution can render it aversive. Therefore, habituation to this fluid is necessary (Figure 1). Previously, habituation was followed by a longer washout period before trauma occurred [20,26]. To shorten the examination period, we first tested whether 3 days’ sucrose habituation (4 h per day, between 9 and 13 h) can influence the effect of trauma on later freezing behavior, as the major measure of PTSD-like symptoms [13,14], started shortly (4 h) after the last habituation.

During all experiments, the mice were traumatized (fear conditioning, FC) 4 h after sucrose habituation in a footshock chamber (Ugo Basile, Gemonio, Italy), then immediately afterwards they were provided either with tap water or different concentrations of sucrose for 24 h/3 h. The animals were returned to the trauma-related context 24 h (recent fear memory)/14 days (remote fear memory) after footshock (conditioned fear test, CFT). In a set of experiments, sucrose solution was provided before the fear conditioning test for 24 h (possible curative effect in contrast to the possible preventive effect, when sucrose drinking started immediately after trauma). A separate set of animals was used for all experiments. Because we had two test chambers, two animals were tested at the same time.

Experiment 1. Effect of habituation. Three groups were compared: non-traumatized controls drinking water during the whole experiment (animals spent the same time in the shock chamber as the traumatized group without trauma), traumatized animals drinking water during the whole experiment, and traumatized mice drinking 16% sucrose for 3 days (daily 4 h) before trauma (habituation) but given water to drink after trauma. CFT was conducted 24 h after FC. Sex differences were also assessed in this study. N = 8/group.

As trauma effectively increased freezing, and habituation had no effect on this parameter, we omitted the non-shock group and used this short habituation.

Experiment 2. Prevention of conditioned freezing 24 h after trauma (ASD-like). Animals were allowed to drink 2%, 16%, or 32% sucrose solution or tap water (controls) for 24 h immediately after trauma, and CFT was conducted 24 h after FC. N = 7–15/group.

Experiment 3. Prevention of conditioned freezing 14 days after trauma (PTSD-like). Animals were allowed to drink 16% or 32% sucrose solution or tap water (controls) for 24 h immediately after trauma, and CFT was conducted 14 days after FC. We omitted 2% sucrose because it had no previous effect. N = 7–18/group.

Experiment 4. Timing of 16% sucrose. As 16% sucrose, administered for 24 h immediately after trauma, was effective in preventing ASD-like freezing in both sexes, we examined whether the first 3 h, suggested by Conoscenti et al. [26], was sufficient to produce the same effect. Thus, Experiment 2 was repeated with 3 h of 16% sucrose drinking in comparison with water drinking. Additionally, we tested whether 16% right before remote CFT can “cure” the PTSD-like behavior. Thus, in this case, 16% sucrose or tap water (controls) was provided for 24 h before the CFT, 14 days after trauma. N = 6–13/group.

### 2.3. Fear Conditioning (FC)

The mice were put into the plastic FC chamber (internal size: 25.5 (d) × 25.5 (w) × 36 (h) cm; Ugo Basile, Gemonio, Italy) through a circular front door. The chamber was housed in sound-attenuating cabinets with white noise (60–70 dB) and was equipped with infrared-sensitive cameras. The behavior was recorded on a laptop using ANY-Maze software (version 7.0; Stoelting Co., Wood Dale, IL, USA). After a 5 min habituation period, a 10 s neutral tone at 3000 Hz and 85 dB was introduced, together with an increase in the light intensity (from 50% to 100%) (together referred to later as cues). During the last 2 s of the cues, a footshock (1.50 mA) was also applied to all stimuli (auditory–visual and footshock), which terminated together. After a further 5 min consolidation period in the FC chamber, the experiment was concluded, and the mice were gently transferred back into their home cages. The box was cleared with tap water and 20% ethanol.

### 2.4. Conditioning Fear Tests (CFTs)

Animals were reintroduced into the FC chamber after 24 h (recent fear memories indicative of ASD-like behavior) and 14 days (remote fear memories suggestive of PTSD-like behavior). We used the same background and cleaning materials as those used in the FC phase. None of the mice were traumatized at this time; however, the cues were applied. Thus, the first 300 s was context-dependent behavior, whereas the last 310 s was cue-related fear memory. All behaviors were blindly analyzed using ANY-Maze software (see earlier).

### 2.5. Statistical Analysis

The data were analyzed by StatSoft 10.0, using mixed ANOVA (sex, sucrose as between-subject factors and context-cue, or day of fluid measurement as repeated factors). The effect size was also calculated (partial eta squared: η^2^_p_) and interpreted as small = 0.01, medium = 0.06, and large = 0.14, according to Cohen [41]. Post hoc comparisons were made using Tukey’s HSD test. Pearson’s correlation was used to search for interactions between the factors. The data were tested for normal distribution and homogeneity of variance and are presented as mean ± SEM. Statistical *p*-value was set at *p* < 0.05.

## 3. Results

### 3.1. Sucrose Habituation

**Figure 2:**
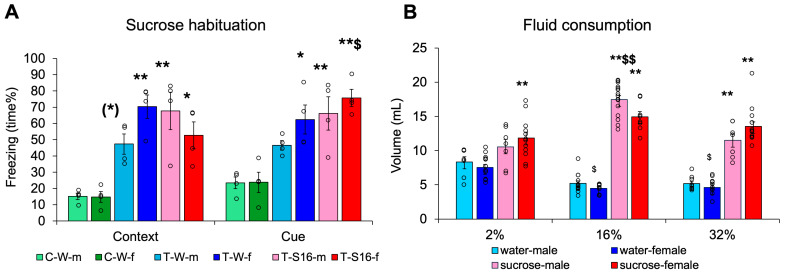
Habituation to high-calorie sucrose drinking. (**A**) Twenty-four hours later trauma increased freezing both in the context as well as after introducing auditory and visual cues. Females were more sensitive to trauma. (**B**) The animals preferred sucrose above water and drank more from 16% sucrose solution than from any other fluid. In general females drank less than males. Abbreviation: C-W-m: control, non-traumatized, water drinking, male; C-W-f: control, non-traumatized, water drinking, female; T-W-m: traumatized, water drinking, male; T-W-f: traumatized, water drinking, female; T-S16-m: traumatized, 16% sucrose drinking, male; T-S16-f: traumatized, 16% sucrose drinking, female; N = 4/group for Figure 2A; for Figure 2B N: water-male 2% 7, 16% 13, 32% 9; water-female 2% 14, 16% 9, 32% 11; sucrose-male 2% 7, 16% 15, 32% 7; sucrose female 2% 12, 16% 8, 32% 15. (*) 0.05 < *p* < 0.10, * *p* < 0.05, ** *p* < 0.01 vs. respective C-W; $ *p* < 0.05, $$ *p* < 0.01 vs. context (Figure 2A) or 2 and 32% sucrose, male (Figure 2B).

First, we confirmed that our intervention could traumatize the animals, as during CFT their freezing behavior increased compared to non-shocked controls (difference between the three treatment groups: F(2,18) = 29.871, *p* < 0.001, η^2^_p_ = 0.768; Figure 2A). Thus, they remembered the trauma. However, the animals remembered the cue better than the context alone (context cue: F(1,18) = 5.036, *p* = 0.038, η^2^_p_ = 0.219; group × context cue: F(2,18) = 4.426, *p* = 0.027, η^2^_p_ = 0.330), and sex also influenced freezing (sex × group × context cue: F(2,18) = 4.504, *p* = 0.026, η^2^_p_ = 0.334). Females spent a greater percentage of time freezing than males, signifying a higher fear response. Although freezing did not correlate with initial body weight, cue-induced freezing was positively correlated with fluid intake (day 1: r = 0.393, *p* = 0.057; day 2: r = 0.551, *p* = 0.005; day 3: r = 0.5964, *p* = 0.002). As sucrose habituation had no effect on the trauma-induced freezing increase, the non-traumatized group was later not involved.

When we measured fluid consumption for 24 h after trauma, three-way ANOVA revealed that the animals drank 16% sucrose more than any other fluid (concentration: F(2,115) = 7.660, *p* < 0.001, η^2^_p_ = 0.118; Tukey HSD post hoc: 16% *p* < 0.001 vs. 2% and 32%), and all mice drank more sucrose than water (fluid type: F(1,115) = 368.899, *p* < 0.001, η^2^_p_ = 0.762), which was not as pronounced in the 2% sucrose drinking group (sex × concentration × fluid type: F(2,115) = 3.411, *p* = 0.0364, η^2^_p_ = 0.056) (Figure 2B). The females drank less during some, but not all, experiments (sex × concentration: F(2,115) = 3.649, *p* = 0.029, η^2^_p_ = 0.060). This might have been attributed to the smaller weight of the females. However, body weight-normalized fluid intake was even higher in females than males (sex: F(1,121) = 5.519, *p* = 0.044, η^2^_p_ = 0.044).

### 3.2. Prevention of Conditioned Freezing 24 h After Trauma by Post-trauma Sucrose Drinking for 24 h

**Figure 3:**
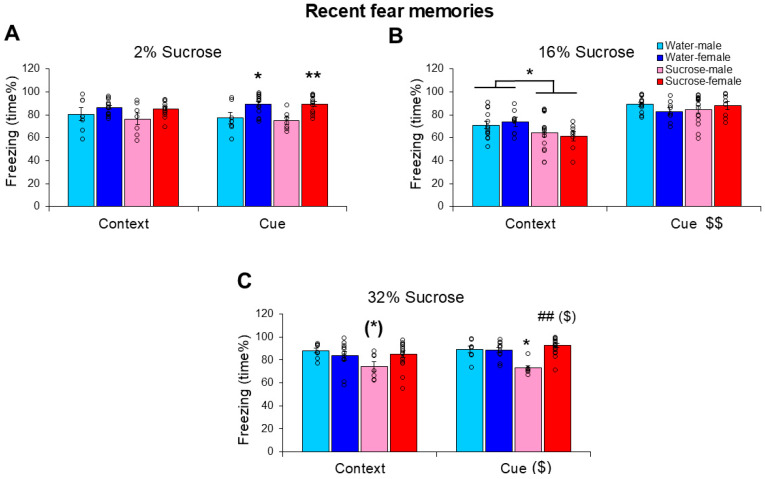
Influence of different sucrose concentrations on recent fear memories resembling acute stress disorder-like behavior. The freezing behavior (time%) of mice 24 h after a trauma was reduced by 16% (**B**) and 32% (**C**), but not 2% (**A**) sucrose drinking between the trauma and conditioned fear testing. The 16% sucrose was similarly effective in both sexes, however, only during the first 5 min (context-dependent phase). While 32% sucrose was effective in males only, the effectiveness was more pronounced on cue-induced freezing. N: water-male 2% 7, 16% 13, 32% 8; water-female 2% 14, 16% 9, 32% 9; sucrose-male 2% 7, 16% 15, 32% 7; sucrose female 2% 12, 16% 8, 32% 7. (*) 0.05 < *p* < 0.10; * *p* < 0.05, ** *p* < 0.05 vs. respective C-W; ## *p* < 0.01 vs. respective male; ($) 0.05 < *p* < 0.10, $$ *p* < 0.01 context vs. cue.

When (after 3 days’ prior habituation) 2% sucrose was administered for 24 h after electric footshock trauma, the treatment did not influence the time the mice spent freezing during recent fear memory testing in the context not in relation to cues (treatment: F(1,36) = 0.522, *p* = 0.475, η^2^_p_ = 0.14; context-cue: F(1,36) = 0.118, *p* = 0.733, η^2^_p_ = 0.003; treatment × context-cue: F(1,36) = 0.281, *p* = 0.599, η^2^_p_ = 0.008) (Figure 3A). In this case, female sensitivity was pronounced (sex: F(1,36) = 14.437, *p* < 0.001, η^2^_p_ = 0.286). The marginal sex × context-cue effect (F(1,36) = 4.021, *p* = 0.052, η^2^_p_ = 0.100) suggested that cues enhanced freezing only in females. Despite being smaller (sex effect on body weight: F(1,36) = 4.300, *p* = 0.044, η^2^_p_ = 0.230), we did not detect sex differences in fluid consumption (F(1,36) = 0.097, *p* = 0.757, η^2^_p_ = 0.003) and there was no interaction between initial body weight and fluid consumption either (r = 0.022, *p* = 0.891). The consumed amount of fluid did not correlate with either the context or cue-induced freezing, even if it was studied separately for water and sucrose groups. Interestingly, the initial body weight was negatively correlated with cue-induced, but not context-induced freezing (r = −0.475, *p* = 0.002).

When 16% sucrose was administered to the mice for 24 h after trauma during recent memory testing, freezing was reduced during the context-dependent phase (treatment × context cue: F(1,41) = 6.362, *p* = 0.016, η^2^_p_ = 0.134) (Figure 3B, Supplementary Table S1). The cue increased freezing compared with context only (F(1,41)= 96.432, *p* < 0.001, η^2^_p_ = 0.702), whereas females were more sensitive to the sucrose effect (sex × treatment × context-cue: F(1,41) = 4.241, *p* = 0.046, η^2^_p_ = 0.094). Pearson’s correlation revealed a positive correlation between sucrose (but not water) drinking and context-dependent freezing (r = 0.426, *p* = 0.042); however, freezing did not correlate with the initial body weight (context: r = −0.043, *p* = 0.777; cues: r = 0.091, *p* = 0.553).

When the sucrose concentration was increased to 32%, the treatment effect became highly significant (F(1,37) = 6.652, *p* = 0.014, η^2^_p_ = 0.074) (Figure 3C). However, the effect of sucrose this time was more pronounced in males (sex: F(1,37) = 4.460, *p* = 0.041, η^2^_p_ = 0.137; sex × treatment: F(1,37) = 9.715, *p* = 0.004, η^2^_p_ = 0.115). Context-cue differences were also observed only in males (sex × context-cue: F(1,37) = 4.313, *p* = 0.045, η^2^_p_ = 0.132), with a more pronounced effect on cue-induced freezing. The consumed amount of fluid did not correlate with either the context or cue-induced freezing, even if it was studied separately for water and sucrose groups. Freezing did not correlate with body weight.

### 3.3. Remote, PTSD-like Memories After Post-trauma Sucrose Drinking

**Figure 4:**
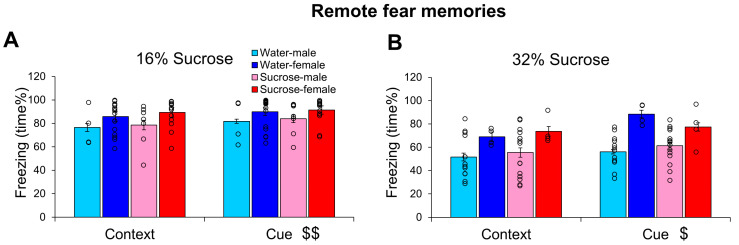
Influence of different sucrose concentration on remote fear memories resembling post-traumatic stress disorder-like behavior. The freezing behavior (time%) of mice 14 days after a trauma was not influenced by 16% (**A**) or 32% (**B**) sucrose drinking for 24 h right after the trauma. Cue induced more freezing than context alone and females were more sensitive to previous trauma. N: water-male 16% 4, 32% 13; water-female 16% 18, 32% 4; sucrose-male 16% 7, 32% 15; sucrose female 16% 15, 32% 4. $ *p* < 0.05, $$ *p* < 0.01 context vs. cue.

When 16% sucrose was provided as a drink immediately after trauma, freezing in the CFT conducted 14 days later was not altered (Figure 4A). The animals spent more time freezing after cues than context only (F(1,40) = 9.688, *p* = 0.003, η^2^_p_ = 0.195), and there was a marginal sex difference (F(1,40) = 3.937, *p* = 0.054, η^2^_p_ = 0.090), with higher freezing in females. Neither the fluid intake nor the initial body weight correlated with freezing behaviors.

A similar effect was observed after 32% sucrose consumption (Figure 4B). Namely, the cue induced stronger freezing than the context alone (F(1,32) = 5.756, *p* = 0.022, η^2^_p_ = 0.152); here, the sex effect was highly significant (F(1,32) = 14.935, *p* < 0.001, η^2^_p_ = 0.318), with females spending more time freezing. Moreover, we did not detect any effect of 32% sucrose consumption either.

### 3.4. Different Timing of 16% Sucrose Drinking

**Figure 5:**
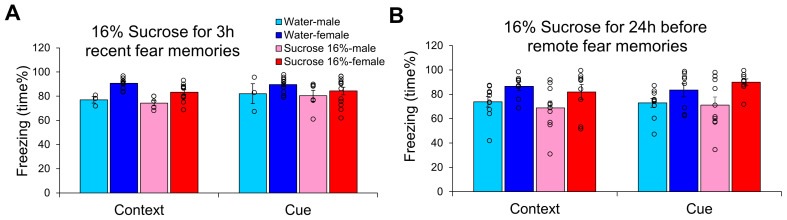
Influence of 16% sucrose drinking on the appearance of conditioned fear. (**A**) When the animals received 16% sucrose for 3 h right after trauma and were tested at 24 h, only the enhanced female sensitivity was detected. (**B**) When 16% sucrose was given for 24 h right before remote conditioned fear test 14 days after trauma, only the sex differences with higher freezing in females were detectable without any effect of the intervention. N: water-male 3 h 3, 24 h 10; water-female 3 h 11, 24 h 8; sucrose-male 3 h 6, 24 h 10; sucrose female 3 h 13, 24 h 9.

When 16% sucrose was provided for 3 h immediately after trauma, there was no change in the time spent freezing 24 h after trauma (Figure 5A). In this case, only the sex effect was significant (F(1,29) = 9.065, *p* = 0.005, η^2^_p_ = 0.238), with higher values in females. Freezing behavior was negatively correlated with fluid intake (context: r = −0.602, *p* < 0.01; cue: r = −0.371, *p* = 0.033), which was attributed to sucrose, but not water drinking (correlation was not detectable with only water, but with only sucrose drinking). Freezing did not correlate with body weight.

In an attempt to cure already-developed fear memories, 16% sucrose was applied for 24 h immediately before remote fear memory testing (Figure 5B). This intervention had no effect on freezing behavior, and the only observable significant effect was the sex difference (F(1,33) = 9.620, *p* = 0.004, η^2^_p_ = 0.226). In this case, both fluid intake (context: r = −0.390, *p* = 0.017) and body weight (context: r = −0.332, *p* = 0.045; cue: r = −0.335, *p* = 0.042) were negatively correlated with freezing.

## 4. Discussion

Our primary finding was that the consumption of 16% sucrose for 24 h post-trauma reduced context-dependent fear memory in both sexes after 24 h (ASD-like) but not after 14 days (PTSD-like). With similar timing, the higher, 32% concentration was effective only in males. Our results have a medium effect size and are consistent with previous rodent investigations [20,26,42] as well as with the anxiolytic effect of sucrose consumption, as observed with up to 15% sucrose in a chronic stress model in male mice [42], and in a human study examining real-world sugar intake [43]. Thus, it may have biological significance. The ineffectiveness of 2% sucrose, in contrast to 16% or 32% sucrose, suggests that low-dose sugar, representing a pleasurable solution with rather low calories, might not be enough to modify the effect of trauma. Previous studies have suggested the need for excessive, high-calorie sugar intake to influence emotional and cognitive processes [20,26,33,44,45]. However, in contrast to a previous study on Sprague-Dawley rats [26], in our study using C57Bl/6 mice, a shorter period (3 h) was not effective in preventing ASD-like behavioral alterations. Thus, even after a short trauma (in our case, ~10 min), prolonged intake from a high-calorie intake might be necessary. We can exclude osmolarity-induced changes (among others, different stomach distension), as the 16% and 32% solutions were similarly effective. A possible explanation for the observed effects might be the more subdued hypothalamic–pituitary–adrenocortical (HPA) axis activation. In line with the comfort food theory, prolonged high-calorie intake, reflected in increased fat storage, is able to diminish corticotropin-releasing hormone expression, the hypothalamic component of the stress axis, leading to reduced stress feeling [32,33,34,46]. Although there is no equivocal increase in stress hormone levels [47], HPA axis dysregulation is definitely present in PTSD [48], which might benefit from high-calorie intake, similar to chronic stress situations [42]. In line with HPA axis dysfunction, high-calorie intake might affect emotional appraisal of the situation, providing a sense of relief and pleasure [49,50]. Moreover, the stimulation of the reward and motivation pathways may provide immediate gratification and reduce the perception of stress, and thus, might further help in coping with trauma [51]. However, when high-calorie intake (i.e., 25% sucrose) starts during early development (i.e., 3 weeks of age), it might have a negative impact on hippocampal neurogenesis and memory formation [52]. Furthermore, consuming too much sugar is increasingly viewed as a risk factor for many chronic diseases such as obesity and dental caries and can even alter hippocampal and amygdala function, key brain regions involved in fear processing [53,54]. Thus, high-calorie food might be good for you immediately after a traumatic event; however, its consumption should be limited to a moderate period.

As the literature is quite inconsistent regarding the nature of HPA axis dysfunction in PTSD [55], we were looking for an alternative explanation of how high-calorie intake might influence trauma-induced freezing. Dietary sucrose may change the microbiota composition of the gut [56], which can lead to neuroinflammation and neurotransmitter imbalance [57]. Previous research has shown that certain changes in the gut microbiome can worsen PTSD symptoms by increasing inflammation and altering neuroactive metabolites [58]. In contrast, microbiota-modifying prebiotic interventions may be helpful in a subset of individuals with PTSD [59]. We might assume that our intervention led to a desirable shift in microbiota composition, providing food for helpful taxa; however, further evidence is needed in this regard. Hyperglycemia is often associated with increased oxidative stress and inflammation [60]. In contrast, when glucose supply is limited and/or in cases of excessive metabolic needs (e.g., strong stressors), glucose is essential for the production of nicotinamide adenine dinucleotide phosphate (NADPH), an important endogenous antioxidant, via the pentose phosphate pathway [61]. Thus, glucose is a major antioxidant immediately after trauma. We may assume that no single mechanism is solely responsible for the acute beneficial effects of high-calorie intake; rather, it is the interplay between multiple factors. Table 1 summarizes the possible mechanisms from previous studies.

In the first experiment, we confirmed that in C57Bl/6 mice, previous habituation to sugar drinking to enhance their compliance had no profound effect on the assessment of fear responses. Interestingly, mice drank more sucrose than tap water, with the highest consumption from the 16% compared to both 2% and 32% sucrose. Our data confirmed previous observations of the 16% sucrose preference of C57Bl/6 mice [37].

Both sexes performed almost equally in terms of the effects of high-calorie fluids on ASD and PTSD symptoms. Notably, female mice appeared to be more sensitive to trauma, as in six out of eight experiments, the sex effect was significant, and in the remaining two experiments, sex influenced the effect of treatment. This is in accordance with the higher prevalence of PTSD in females [39] as well as their increased stress hormone levels and stress sensitivity attributed to gonadal hormones [63]. The observation that females exhibited heightened sensitivity to the 16% sucrose treatment, whereas males demonstrated increased sensitivity to 32% sucrose, may suggest sex-specific differences in stress coping and reward processing. Previous studies in rats suggested that circulating estrogen may lower detectability thresholds for sweet stimuli [64], supported by a human study, indicating that males prefer higher stimulus levels, whereas women show less preference for very high sweetness [65]. Variations in hormonal levels, metabolic processes, or neural circuitry may account for these sex differences, which in turn affect PTSD-like fear responses.

Our interventions (3 × 4 h habituation and/or sucrose consumption for a maximum of 24 h) had no effect on body weight changes. Although the expected sex differences were observable females being smaller, body weight correlated with freezing only in two series, suggesting that the increased trauma sensitivity of female mice is not due to their smaller weight. Indeed, no literature data were found on the correlation between initial body weight and freezing, despite clear evidence that in rats, trauma induced smaller weight gain with enhanced freezing compared with non-traumatized animals [66]. Similarly, the amount of fluid consumed after trauma had no clear correlation with freezing behavior (positive, negative, and no correlation detected). We might have expected a negative correlation, as fear conditioning was reported to reduce reward-seeking behavior and consuming sweet solutions might be considered as a reward [67,68]. Thus, our assumption was that the higher the freezing (reflecting stronger fear conditioning), the lower the amount of sucrose consumed. Interestingly, only 16% sucrose consumption showed any correlation with freezing, further confirming that this concentration was preferred by this mouse strain. Lower than 16% might not provide enough energy, while higher concentrations might be osmotically more demanding, stressful, and therefore more aversive than rewarding. Thus, there is a fine balance between the harmful and beneficial effects of high calorie intake (e.g., pro- and antioxidative effects [61]), with an optimum at this concentration.

### 4.1. Strengths of Our Project

Many studies predominantly used male mice, despite the higher prevalence of PTSD in females [69]. Our approach addresses this gap by utilizing a short, well-defined trauma model with commercially available equipment to ensure greater reliability and reproducibility. Additionally, by working with laboratory animals under well-controlled conditions, we can minimize the variability observed in human populations, such as differences in genetic background, environmental factors, and nutrition, further supporting reliability.

The gut microbiome, a key player in glucose uptake, shares a high degree of similarity between mice and humans [70], strengthening the translational value of our study. Importantly, we not only investigated ASD-like changes, but also examined a later time point after trauma, which may correspond to PTSD in humans. This aligns with the DSM-5-TR criteria, which emphasize the prolonged presence of symptoms as a defining characteristic of PTSD [71].

### 4.2. Limitations

Although footshock and other electroshock methods are widely used in animal models, they are relatively uncommon causes of PTSD in humans. Additionally, while fear conditioning in animals occurs over a matter of minutes, human trauma can range from a moment to an extended period, spanning several weeks.

Our study primarily focused on freezing behaviour as an indicator of memory retention; however, PTSD encompasses a broader spectrum of symptoms beyond this measure [12,72]. Furthermore, the habituation period ended four hours before trauma exposure, which may have influenced the outcomes. Moreover, animals were isolated before testing to follow and control their individual fluid intake, which can be considered an additional stress factor. Indeed, prolonged (minimum of 6 weeks) social isolation may alter freezing behavior in mice, with some authors reporting an increase [73], while others report a decrease [74], and no difference was found after 3–4 weeks [38]. Thus, we believe that the short (less than one week) isolation in our case did not significantly modify the observed changes. Nonetheless, all animals, including those subjected to later water consumption, were habituated to sucrose and housed individually, minimizing the likelihood of group differences arising from these factors.

## 5. Conclusions

Our results support the short-term anecdotic psychological benefits of high-calorie intake in humans, which is an easy method for temporary emotional relief. Unfortunately, this intervention is unlikely to have a sustainable therapeutic effect in PTSD. Moreover, individual differences might exist in the optimal dose/concentration, which should be considered. Overall, these results emphasize the complexity of the relationship among sucrose consumption, sex differences, and memory recall in response to trauma, warranting further exploration of the mechanisms underlying these effects.

## Figures and Tables

**Table 1:** Summary of the possible mechanism.

Mechanism	Impact of High Calorie on ASD	References
Meets the increased energy demand	Stabilizes metabolic responses, reduces acute stress	[20,26,33,44,45]
HPA axis dampener	Provides negative feedback to the HPA, thus, reducing glucocorticoid secretion	[32,34,62]
Emotional–cognitive regulator	Alters emotional appraisal of the situation, provides psychological comfort, reduces anxiety by stimulating reward and motivation	[42,49,50,51]
Has beneficial impact on the gut microbiome	Shifts microbiota composition to reduce neuroinflammation	[59]
Antioxidant	NADPH production via the pentose phosphate pathway	[61]

## Data Availability

The data are available upon reasonable request.

## Supplementary Materials

The following supporting information can be downloaded at: https://www.mdpi.com/article/10.3390/biomedicines13092233/s1, Table S1: Raw data for 16% sucrose drinking (posttrauma, 24h). The 5. column contains time spent in freezing during context presentation (in %), while the 6. column contains time spent freezing to tone-light cues (in %).

## Abbreviations

The following abbreviations are used in this manuscript:

ASD	Acute stress disorder
CFT	Conditioned Fear Test
FC	Fear conditioning
HPA	Hypothalamic–pituitary–adrenocortical
NADPH	Nicotinamide adenine dinucleotide phosphate
PTSD	Post-traumatic stress disorders
