# Supplementary material for: Sweet Relief? Short-Term Post-Traumatic High-Sucrose Intake Attenuates Acute but Not Long-Term Fear Responses in Mice
Source: Biomedicines. 2025 Sep 11;13(9):2233. doi: 10.3390/biomedicines13092233 (PMC12467851; doi:10.3390/biomedicines13092233)
Supplement: Supplementary file 1 [file biomedicines-13-02233-s001.zip › biomedicines-3807701-supplementary.pdf]

Series	Animal ID	Age	Gender	Treatment	nce Before	between Sh	CFT conten	CFT with
1	1.	4	Male	Sucrose	101,6	101,6	203,1	259,3
1	3.	4	Male	Sucrose	101,1	104,0	250,6	241,2
1	5.	4	Male	Sucrose	98,0	106,9	217,9	203,2
1	7.	4	Male	Sucrose	100,0	98,7	104,6	139,4
1	9.	4	Male	Sucrose	101,4	99,3	97	202,6
1	11.	4	Male	Sucrose	105,7	100,0	80,9	98,3
1	13.	4	Male	Sucrose	106,5	96,7	252,8	163,5
1	15.	4	Male	Sucrose	99,1	100,9	83,6	180
1	17.	4	Male	Sucrose	103,8	103,7	225,8	171,4
1	19.	4	Male	Sucrose	110,1	96,9	138,9	232,6
1	2.	4	Male	Water	106,3	102,0	210,8	190,9
1	4.	4	Male	Water	101,2	103,3	126,9	151,5
1	6.	4	Male	Water	103,1	103,4	155,4	184,6
1	8.	4	Male	Water	101,8	98,6	155,2	147,4
1	10.	4	Male	Water	100,7	98,6	112	178,2
1	12.	4	Male	Water	97,2	107,0	137,1	158,7
1	14.	4	Male	Water	104,1	100,0	221,5	203,1
1	16	4	Male	Water	104,6	100,0	112,1	232,5
1	18.	4	Male	Water	106,0	95,3	253,5	208,9
2	5.	6	Female	Sucrose	105,2	101,7	208,4	251,2
2	7.	6	Female	Sucrose	103,1	101,0	203,2	173,5
2	9.	6	Female	Sucrose	106,0	96,5	197,3	234,3
2	11.	3	Female	Sucrose	104,7	96,0	275,3	300,4
2	1.	3	Male	Sucrose	101,5	101,1	139,0	208,7
2	3.	3	Male	Sucrose	101,8	97,9	112,9	200,1
2	13.	3	Male	Sucrose	99,7	99,0	195,9	192,5
2	15.	3	Male	Sucrose	102,9	98,1	202,8	236,4
2	17.	3	Male	Sucrose	97,3	99,1	190,6	121,1
2	6.	6	Female	Water	106,1	93,5	184,9	297
2	8.	6	Female	Water	105,5	95,2	192,2	298,6
2	10.	6	Female	Water	106,4	98,7	229	257,4
2	12.	3	Female	Water	100,5	100,0	221,6	244
2	2.	3	Male	Water	103,2	101,2	86,6	146,6
2	4.	3	Male	Water	101,0	97,7	218,1	240
2	14.	3	Male	Water	100,3	96,4	131	114,6
2	16	3	Male	Water	78,9	101,4	92,3	103,1

CFT conten	CFT with	ke 24 hr. Af
67,7	83,6	15,7
83,5	77,8	15,3
72,6	65,5	10,4
34,9	45,0	15,1
32,3	65,4	13,9
27,0	31,7	13,6
84,3	52,7	16
27,9	58,1	12,8
75,3	55,3	17,3
46,3	75,0	18
70,3	61,6	6
42,3	48,9	6,2
51,8	59,5	5,5
51,7	47,5	6,1
37,3	57,5	6,1
45,7	51,2	4,9
73,8	65,5	6
37,4	75,0	7,3
84,5	67,4	9,8
69,5	81,0	14,8
67,7	56,0	12,8
65,8	75,6	13,5
91,8	96,9	14,3
46,3	67,3	13,6
37,6	64,5	15,3
65,3	62,1	18,6
67,6	76,3	16,1
63,5	39,1	19,2
61,6	95,8	5,4
64,1	96,3	5,2
76,3	83,0	5,2
73,9	78,7	4,9
28,9	47,3	6,6
72,7	77,4	5,7
43,7	37,0	8,9
30,8	33,3	8,3
